# The Potential of Evidence-Based Clinical Intake Tools to Discover or Ground Prevalence of Symptoms Using Real-Life Digital Health Encounters: Retrospective Cohort Study

**DOI:** 10.2196/49570

**Published:** 2024-07-16

**Authors:** Eden Avnat, Michael Samin, Daniel Ben Joya, Eyal Schneider, Elia Yanko, Dafna Eshel, Shahar Ovadia, Yossi Lev, Daniel Souroujon

**Affiliations:** 1 Kahun Medical Ltd Givatayim Israel; 2 Faculty of Medicine Tel-Aviv University Tel Aviv Israel; 3 Faculty of Health Sciences Ben-Gurion University of the Negev Beer Sheva Israel; 4 Department of Otolaryngology-Head and Neck Surgery Samson Assuta Ashdod University Hospital Ben Gurion University Faculty of Health Sciences Ashdod Israel; 5 The Azrieli Faculty of Medicine Bar-Ilan University Safed Israel

**Keywords:** clinical intake tool, evidence-based medicine, big data, digital health, symptoms, prevalence

## Abstract

**Background:**

Evidence-based clinical intake tools (EBCITs) are structured assessment tools used to gather information about patients and help health care providers make informed decisions. The growing demand for personalized medicine, along with the big data revolution, has rendered EBCITs a promising solution. EBCITs have the potential to provide comprehensive and individualized assessments of symptoms, enabling accurate diagnosis, while contributing to the grounding of medical care.

**Objective:**

This work aims to examine whether EBCITs cover data concerning disorders and symptoms to a similar extent as physicians, and thus can reliably address medical conditions in clinical settings. We also explore the potential of EBCITs to discover and ground the real prevalence of symptoms in different disorders thereby expanding medical knowledge and further supporting medical diagnoses made by physicians.

**Methods:**

Between August 1, 2022, and January 15, 2023, patients who used the services of a digital health care (DH) provider in the United States were first assessed by the Kahun EBCIT. Kahun platform gathered and analyzed the information from the sessions. This study estimated the prevalence of patients’ symptoms in medical disorders using 2 data sets. The first data set analyzed symptom prevalence, as determined by Kahun’s knowledge engine. The second data set analyzed symptom prevalence, relying solely on data from the DH patients gathered by Kahun. The variance difference between these 2 prevalence data sets helped us assess Kahun’s ability to incorporate new data while integrating existing knowledge. To analyze the comprehensiveness of Kahun’s knowledge engine, we compared how well it covers weighted data for the symptoms and disorders found in the 2019 National Ambulatory Medical Care Survey (NMCAS). To assess Kahun’s diagnosis accuracy, physicians independently diagnosed 250 of Kahun-DH’s sessions. Their diagnoses were compared with Kahun’s diagnoses.

**Results:**

In this study, 2550 patients used Kahun to complete a full assessment. Kahun proposed 108,523 suggestions related to symptoms during the intake process. At the end of the intake process, 6496 conditions were presented to the caregiver. Kahun covered 94% (526,157,569/562,150,572) of the weighted symptoms and 91% (1,582,637,476/173,4783,244) of the weighted disorders in the 2019 NMCAS. In 90% (224/250) of the sessions, both physicians and Kahun suggested at least one identical disorder, with a 72% (367/507) total accuracy rate. Kahun’s engine yielded 519 prevalences while the Kahun-DH cohort yielded 599; 156 prevalences were unique to the latter and 443 prevalences were shared by both data sets.

**Conclusions:**

ECBITs, such as Kahun, encompass extensive amounts of knowledge and could serve as a reliable database for inferring medical insights and diagnoses. Using this credible database, the potential prevalence of symptoms in different disorders was discovered or grounded. This highlights the ability of ECBITs to refine the understanding of relationships between disorders and symptoms, which further supports physicians in medical diagnosis.

## Introduction

Evidence-based medicine (EBM) has become an integral part of modern medical practice [1]. It relies on the use of systematic research to identify up-to-date and reliable data, which serves as a crucial component in making medical decisions for individual patients [2]. The use of EBM can help reduce outdated practices or implicit biases that may disrupt medical decision-making such as prioritizing White individuals over minorities in emergency care or underestimating diagnosis of coronary heart disease in females [3-5].

The emergence of the big data revolution in the medical world not only reduces some of the bias and keeps physicians updated, but also serves as an important factor in the evolution of EBM [6-8]. By using machine learning tools, implicit insights can be more easily derived from raw data, and instead of focusing on data analysis as a tool to answer questions, it is now used as a tool to find new questions which can lead to new and promising hypotheses [7].

In particular, personalized medicine has benefited from the analysis of abundant and diverse data [9]. Analyzing multi-omics data derived from large-scale cohort and population studies, combined with the study’s conclusions, allows for identifying subtle differences in an individual’s genetics which may lead to precise and personalized interventions [9].

Today, as the demand for personalized medicine based on the EBM approach is on the rise, health providers are seeking to bridge the gap between the EBM paradigm which formulates generalized conclusions gathered from many studies, and personalized medicine that focuses on the individual [10,11]. One solution that bridges that gap suggests that using evidence-based clinical intake tools (EBCITs), such as Kahun [11,12], can help physicians with more personalized decision-making.

Kahun is an artificial intelligence engine, encompassing more than 50,000 peer-reviewed publications and more than 30,000,000 medical relations and insights that were mapped to a knowledge graph [13]. Using its evidence-based knowledge graph, Kahun asks the patient personalized questions and then uses dynamic reasoning to generate tailored clinical assessments [12,13].

Prior studies have focused primarily on the diagnostic accuracy of Kahun and other diagnostic support tools [12,14-16]. However, one study [17] that examined COVID-19 patients and their specific symptoms showed that these tools can also provide medical insights such as extending the understanding of symptoms associated with a specific disease such as COVID-19. By collecting patient findings and diagnoses, EBCITs have the potential to become comprehensive real-world databases in their own right. Analyzing these databases using big data methods could contribute to medical knowledge by grounding the real prevalence of symptoms in medical conditions or identifying new ones while supporting the EBM approach.

This study aims to test whether EBCITs cover symptoms and diagnoses similar to physicians and thus serve as a potential reliable tool for decision-making in the clinic. Furthermore, this study demonstrated the ability of EBCITs to identify current or emerging prevalence of symptoms in disorders, thereby enhancing medical knowledge and providing additional support for physicians in clinical practice.

## Methods

### Study Design and Data Collection

This study includes the analysis of 3 data sets.

#### Kahun’s Knowledge Engine

As mentioned, Kahun’s data are based on more than 30,000,000 medical relations and insights from over 50,000 peer-reviewed publications that were mapped by medical experts to a knowledge graph [13]. The medical knowledge is represented by the nodes and the edges of the knowledge graph. For example, node1 represents a specific disorder, which is mapped by edge1 to node2, which represents a specific symptom. The data on edge1 represents the relationship between node1 and node2 such as prevalence. Therefore, the triple (node1, edge1, and node2) represents the prevalence of the specific symptom in the specific disorder. These data are referred to here as Kahun’s knowledge engine.

#### Kahun-Digital Health Care Cohort

This data set was collected by the Kahun platform which assessed patients who received digital health care (DH) services from a DH provider based in the United States, specializing in doctor-patient medical visits that occur using camera-enabled smartphones or computers, between August 1, 2022, and January 15, 2023.

Patients who used the provider’s services were first given a link for initial assessment by Kahun. During the Kahun assessment, questions regarding the patient’s medical background, chief complaint, symptoms, and risk factors were personally generated based on the Kahun algorithms [12]. Additionally, during each assessment, the algorithm suggested a dynamic list of relevant differential diagnoses (disorders) while computing a matching probability between the findings and each disorder. All the data and metadata regarding questions, answers, and disorders were anonymized and stored in a separate part of the Kahun database, which is not related to Kahun’s knowledge engine and is referred to here as the Kahun-DH cohort.

#### National Ambulatory Medical Care Survey

The data were obtained from the latest (2019) National Ambulatory Medical Care Survey (NAMCS), which was conducted by the Centers for Disease Control and Prevention [18]. NAMCS 2019 was designed to provide objective information about the provision and use of ambulatory medical care services in the United States [18]. The findings in NAMCS are based on a sample of weighted visits to nonfederally employed office-based physicians, who are primarily engaged in direct patient care. The findings included “reason of visit #1-5” (coded by NAMCS internal method), “diagnosis #1-5” (*ICD-10* [*International Statistical Classification of Diseases, Tenth Revision*] coded), and more. Since the scope of this study covered symptoms and disorders, we addressed only NAMCS symptoms related to the “SYMPTOM MODULE” and only those disorders that appeared among the NAMCS diagnoses. Findings were excluded where the reason for the visit was related to follow-up visits and the prescription of medication or diagnoses related to an encounter for a specific examination.

### Study Population

Kahun-DH cohort included patients aged 16 years or older who used the provider’s services and completed the assessment by Kahun. Assessments missing information regarding sex, chief complaint, and differential diagnosis were excluded.

### Analysis and Variables

All statistical analyses were performed using RStudio (R version 4.2.2; Posit PBC). Categorical variables were represented by percentage while continuous variables were represented by mean and SD values if distributed normally, and otherwise median and IQR values. A “positive symptoms ratio” was determined as the ratio between the total number of new symptoms or refinements of known symptoms the patient confirmed during the assessment, and the total number of symptoms or refinements suggested to the patient during the assessment. “Total questions” included only those questions that were answered by the patients during the assessment.

### Kahun’s Coverage Rates

We calculated how well Kahun covers NAMCS data by multiplying the ratio of symptoms or disorders that were reported in each visit and appeared in Kahun’s knowledge engine, with the relevant visit weighted score designated by NAMCS. All multiples were summed and then divided by the summed total of all the visits’ weighted scores. We calculated the coverage ratio for the entire set of symptoms and disorders, for the groups of symptoms subset by NAMCS’s “SYMPTOM MODULE,” and for groups of disorders classified according to *ICD-10* prefixes.

### Kahun’s Diagnostic Ability

To evaluate Kahun’s diagnostic ability, 250 sessions (10% of all sessions) were randomly selected and blindly assessed by Israeli licensed physicians. The full transcript of Kahun’s sessions, including questions suggested by Kahun and answers provided by the patient, was given to the physicians. The transcript did not include Kahun’s suggestions for differential diagnosis. Then, based on the transcript received, the physicians suggested up to 3 (nonordered) suitable diagnoses. The physicians’ suggested diagnoses were compared with both the relevant diagnoses and the relevant number of diagnoses suggested by Kahun (eg, if only 2 diagnoses were suggested by the physicians, only 2 diagnoses suggested by Kahun were compared). Kahun’s accuracy rate was set as the number of matched diagnoses suggested by the physicians and Kahun divided by the total number of diagnoses suggested by Kahun (eg, in a specific session, if Kahun suggested 3 different disorders and 2 of them were also suggested by the physician, Kahun’s accuracy rate was 67%, 2/3).

### Prevalence

Kahun calculated the prevalence of symptoms in different disorders based on the data from Kahun’s knowledge engine and independently, based on the Kahun-DH cohort. For each disorder, symptoms that were suggested in more than 29 different assessments were selected. The prevalence of a symptom in a disorder was determined by the ratio of the total occurrences of the confirmed symptom to the total occurrences of the suggested symptom.

Only disorders and symptoms that appeared in both data sets were included. This created 2 equally dimensioned prevalence matrices: the Kahun prevalence matrix and the Kahun-DH cohort prevalence matrix. The prevalence similarity ratio was determined by dividing a prevalence from Kahun’s prevalence matrix with the corresponding prevalence extracted from the Kahun-DH cohort’s prevalence matrix.

Each prevalence matrix underwent hierarchical clustering that clustered disorders by symptoms using the pheatmap R package [19].

### Ethical Considerations

This study analyzed anonymized data from 3 sources: Kahun’s knowledge engine (published peer-reviewed papers), the Kahun-DH cohort, and the NAMCS. The data used in the Kahun-DH cohort was anonymized and deidentified according to HIPAA (Health Insurance Portability and Accountability Act) safe-harbor privacy rules. The data from NAMCS is publicly available and has been anonymized by the Centers for Disease Control and Prevention. As such, this study did not require Institutional Review Board approval, ethical review, or individual informed consent—all the data used was anonymized and deidentified, with no risk to individual privacy. Additionally, no personal or identifying information about participants was accessed or stored during the study. Appropriate measures were taken to ensure compliance with relevant privacy guidelines. No compensation was provided to participants, as this study was a secondary analysis of existing anonymized data sets.

## Results

### Kahun’s Coverage Rates (Kahun’s Knowledge Engine vs NAMCS)

**Figure 1:**
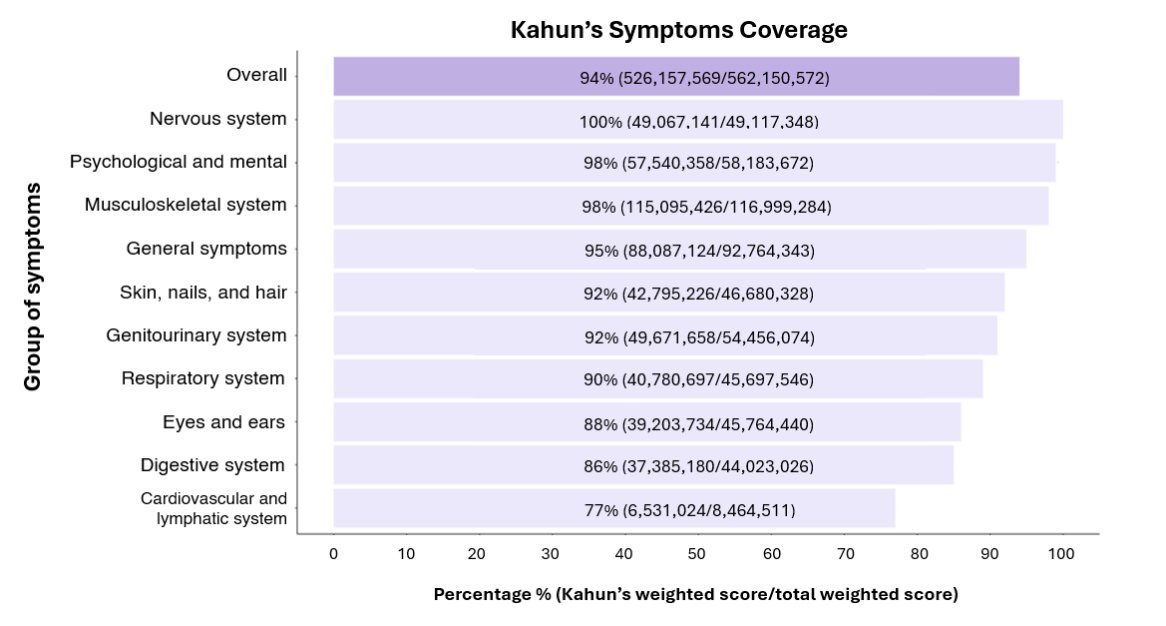
Kahun’s coverage rates for weighted symptoms reported in the National Ambulatory Medical Care Survey 2019. Purple-colored bars represent total symptoms while light-purple bars represent different groups of symptoms.

**Figure 2:**
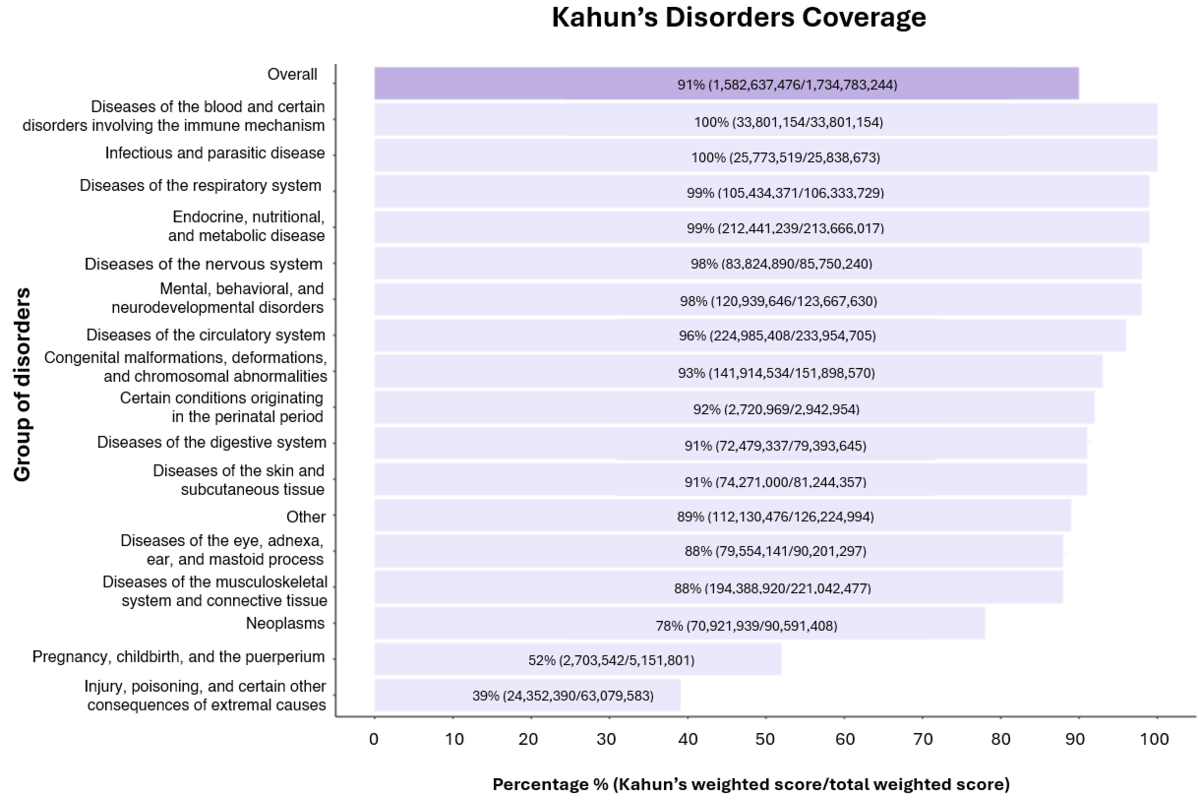
Kahun’s coverage rates for weighted disorders reported in the National Ambulatory Medical Care Survey 2019. Purple-colored bars represent total disorders while light-purple bars represent different groups of disorders.

Kahun covered 94% (526 million/562 million) of all weighted symptoms reported in the NAMCS 2019 data set. In 9 out of 10 different symptom groups, there was coverage for at least 85% of weighted symptoms. Notably, there was nearly complete coverage in the nervous system group (Figure 1). Additionally, Kahun covered 91% (1582 million/1734 million) of all weighted disorders reported in the NAMCS 2019 data set, with at least 88% coverage for weighted disorders in 14 out of 17 different disorder groups. Interestingly, nearly complete coverage was observed for diseases of the blood and certain disorders involving the immune mechanism, and for infectious and parasitic diseases groups (Figure 2).

### Patients’ Characteristics (Kahun-DH Cohort)

Kahun-DH cohort included 1714 women (67%) and 836 men (33%) with a median age of 35 (IQR 16-90) years (Table 1). During each session, a median of 34 (IQR 11-61) questions were asked with a positive symptom ratio of 0.3 (Table 1).

During the assessments, 314 unique chief complaints were reported. Among them, the 5 most frequent chief complaints were anxiety (n=173), sore throat (n=153), cough (n=138), sinus pain (n=114), and headache (n=108). Additionally, the duration of the most frequent chief complaints was between 24 hours and 1 week for 32.3% (836/2550) of the complaints (Table 1).

Overall, Kahun generated 108,523 suggestions relating to 905 unique symptoms and provided 6496 disorders (321 unique disorders) as possible diagnoses.

**Table 1:** Patients’ characteristics.

Characteristics			Overall (N=2550)
**Age (years)**			
	Mean (SD)	38.3 (14.3)	
	Median (IQR)	35.0 (16.0-90.0)	
**Sex, n (%)**			
	Female	1714 (67.2)	
	Male	836 (32.8)	
**Duration of chief complaint, n (%)**			
	<24 hours	325 (12.7)	
	>24 hours and <1 week	824 (32.3)	
	>1 week and <12 weeks	481 (18.9)	
	>12 weeks	344 (13.5)	
	Missing	576 (22.6)	
**Total questions**			
	Mean (SD)	33.6 (9.20)	
	Median (IQR)	34.0 (11.0-61.0)	
**Positive symptoms ratio**			
	Mean (SD)	0.322 (0.201)	
	Median (IQR)	0.297 (0.0244-1.00)	

### Kahun’s Diagnostic Ability (Kahun-DH Cohort)

A random sample of 250 Kahun-DH sessions was selected to assess Kahun’s diagnostic ability. The sessions were blindly evaluated by physicians. In 90% (224/250) of the sessions, at least one identical disorder matched the differential diagnosis suggested by the physicians and the differential diagnosis suggested by Kahun. Additionally, 367 diagnoses that were suggested by Kahun matched the diagnoses suggested by the physicians, resulting in a 72% (367/507) accuracy rate.

### Prevalence (Kahun-DH Cohort vs Kahun’s Knowledge Engine)

**Figure 3:**
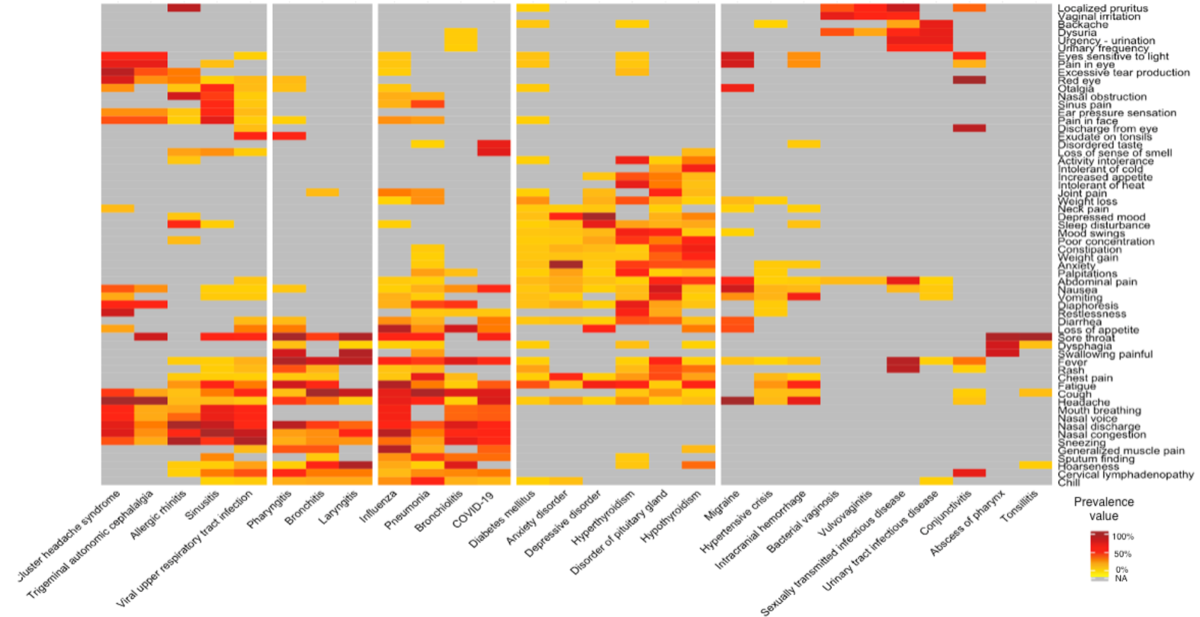
Heat map of the prevalence matrix of symptoms in disorders based on Kahun’s knowledge engine. The white vertical lines cluster the disorders by their symptoms-prevalences distribution, using the hierarchical clustering algorithm. Dark red colors represent high prevalence while light yellow colors represent low prevalence. Gray squares represent symptoms that were not suggested by Kahun during sessions dealing with the corresponding disorder.

**Figure 4:**
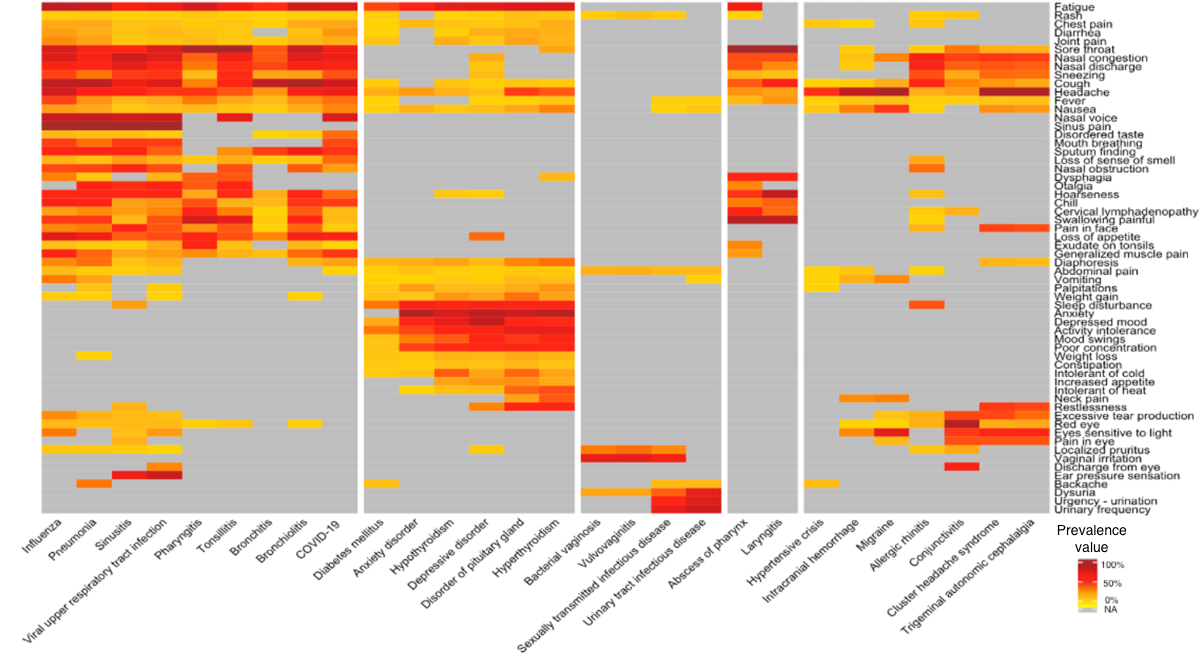
Heat map of the prevalence matrix of symptoms in disorders, based on Kahun-DH cohort. The white vertical lines cluster the disorders by their symptoms-prevalences distribution, using the hierarchical clustering algorithm. Dark red colors represent high prevalence while light yellow colors represent low prevalence. Gray squares represent symptoms that were not suggested by Kahun during sessions dealing with the corresponding disorder. DH: digital health care.

Kahun calculated the prevalence of 60 symptoms in 28 disorders. A total of 519 prevalences were detected based on Kahun’s knowledge engine, whereas 599 prevalences were detected based on the data from the Kahun-DH cohort (Figures 3 and 4, respectively). There was no statistically significant difference between the median prevalence value of Kahun’s knowledge engine and the Kahun-DH cohort: 21% (IQR 5%-50%) and 23% (IQR 8%-47%), respectively (Wilcoxon *P*=.35).

Out of the prevalences that originated in the Kahun-HV cohort, 159 did not appear in Kahun’s knowledge engine. This resulted in a median detection rate of 4 (IQR 1-10) new prevalences per disorder. Tonsillitis had the highest detection rate with 24 new prevalences, while 6 disorders had no new prevalences: disorders of the pituitary gland, hypertensive crisis, hypothyroidism, laryngitis, sexually transmitted infection diseases, and urinary tract infection diseases. Additionally, 443 prevalences were identified in both Kahun’s knowledge graph and the DH patient cohort with a median prevalence similarity ratio of 1.04 (IQR 0.61-2.27). Among them, the prevalence similarity ratio of 85 (19%) prevalences ranged from 0.85 to 1.15.

Using a hierarchical clustering algorithm, 5 clusters of disorders were established based on their symptoms prevalence. Disorders with relatively similar symptom prevalence distribution were clustered together. The prevalence of symptoms that were not suggested by Kahun during the sessions in the corresponding disorder, though suggested in other disorders, was not calculated. The median number of disorders per cluster in Kahun’s knowledge engine was 5, whereas the median number of disorders per cluster was 6 in the Kahun-DH cohort (Figures 3 and 4).

## Discussion

### Principal Findings

This study tested the potential of EBCITs to cover the same medical data assessed by physicians, thus acting as a reliable source for drawing medical conclusions in addition to identifying or grounding possible prevalences of symptoms in different disorders. We used the data in the Kahun EBCIT to examine our hypothesis.

Kahun’s relatively comprehensive coverage of the weighted symptoms collected by NAMCS 2019 shows that although Kahun is not a human physician, it can address at least 94% of the actual symptoms that patients report during medical visits. Moreover, when the symptoms are classified into groups, Kahun still covers most of the symptoms in each group. Kahun also generates differential diagnoses while taking into account about 91% (1,582,637,476/1,734,783,244) of the weighted disorders that physicians diagnosed in NAMCS 2019. These encouraging coverage rates indicate that data gathered by Kahun is almost identical to the data gathered by a human physician, and therefore, relatively reliable.

Moreover, while compared with human medical licensed physicians, Kahun’s decent diagnostic ability indicates that the differential diagnosis suggested by Kahun and therefore its reasoning and intake process are reliable and trustable. Hence, we can infer that medical insights and assessments based on this data are valid.

To identify the prevalence of symptoms in disorders, we analyzed the Kahun-DH cohort. Similarly to other remote-care service users, most of the patients in this cohort were females and relatively young. Additionally, the total number of questions that were asked during the assessment was similar to other clinical intake tools [20]. Comparing a real physician interview and EBTICs might reveal a difference in the effectiveness of these approaches and should be explored further. While focusing on chief complaints, this study highlighted frequent chief complaints that are considered common among primary physicians, as suggested by other works [21,22]. This supports the claim that the data in the study agrees with real-world data. Additionally, more than 40% of the chief complaints lasted less than a week, presenting the relative acute characteristic of the symptoms, which although not widely studied, are supported by Lee et al [23].

The relatively young age of the patients and the fact that those patients were assessed by the DH’s physicians only via digital encounters, might indicate that some of the typical brick-and-mortar clinic patients in the traditional setting of primary care are underrepresented in this cohort. Although telemedicine and digital encounters are gaining more popularity worldwide [24], we believe that evaluating EBCITs in a typical primary clinic setting is also important and should be considered in future research.

Nearly 109,000 suggestions, relating to 905 unique symptoms and approximately 6500 diagnoses of 321 distinct disorders, were included in this study, demonstrating the abundance of data gathered. Having such diverse data could enable profound research.

Moreover, the Kahun-DH cohort included only 2550 patients. Thus, increasing the use of Kahun, which was shown to have decent diagnostic accuracy [12], has the potential to assist physicians in medical assessment while contributing to extended research by enriching Kahun’s database even further.

This study focused on the prevalence of symptoms in disorders. We compared the prevalences generated by Kahun’s knowledge engine with prevalences from the Kahun-DH cohort. We found 156 prevalences, exclusively in the latter group. These unique prevalences emphasize that even after learning and memorizing more than 50,000 medical papers, just as Kahun did [13], medical knowledge can still be improved. Thus, consistently incorporating real-world data into EBCITs, like Kahun, could result in more precise and effective diagnoses which may help physicians in medical decision-making. An example of this assumption is partly demonstrated by the relative medical-improvement in the hierarchical clustering of the 28 analyzed disorders. The hierarchical clustering algorithm grouped together disorders with similar symptoms-prevalence distributions, which may resemble physicians’ process of medical reasoning. For example, according to Figure 4, bacterial vaginosis, vulvovaginitis, sexually transmitted infection diseases, and urinary tract infection diseases were clustered together as they shared similar symptoms-prevalence distribution and can reasonably be clustered as genital-related clusters. By comparing the suggested clusters of the disorders, as shown in Figures 3 and 4, sexually transmitted infection diseases and headache-related disorders were reclustered with more appropriate disorders for each, as noted by certificated physicians. This improvement may indicate that as EBCITs are being used more, they result in better performances, thus suggesting more relevant diagnoses to the physician.

By assessing patients while taking into account a wide range of differential diagnoses and systematically storing the data, Kahun can ask unorthodox questions and less obvious prevalences may be discovered. These prevalences have the potential to serve as new leads for innovative research or medical trends. For example, a new possible prevalence of hoarseness in pharyngeal abscesses was identified by the Kahun-DH cohort, with an estimated prevalence value of 48% (19/40). Literature reviews suggest that such a prevalence exists [25-27]; although, as far as we know, an exact evaluation of this prevalence was not conducted. Using the Kahun-DH cohort, we were able to estimate this prevalence.

A practical example of the potential of EBCIT in grounding prevalences is the prevalence of nasal congestion in migraines. Although such a prevalence did not appear in Kahun’s knowledge engine, according to the data from the Kahun-DH cohort, the prevalence was 28% (9/32; Figure 4). Remarkably, a study by Muehlberger et al [28] evaluated this prevalence as 25%. These relatively close estimations testify to Kahun’s potential to discover prevalences accurately.

Apart from the novel prevalences that were discovered, 443 prevalences were simultaneously identified in both data sets. Of those prevalences, 81% had relative difference values (prevalence similarity ratio >±0.15). This crucial finding highlights the importance of grounding prevalences. By combining comprehensive databases, such as the ones EBCITs may provide, with the information from previous studies, these discrepancies could be resolved and more refined prevalences would be computed. A different approach for settling these discrepancies argues that each cohort represents a different population. Hence, each prevalence is valid as long it refers to its original cohort. The latter approach may be helpful in creating personalized medical prevalences.

While comparing Kahun-DH’s prevalences with Kahun’s knowledge engine prevalences, we included only selected symptoms and disorders, mainly to compare reasoning and sample size issues. Other prevalences were identified based on Kahun’s knowledge engine and the Kahun-DH cohort but are not presented in this analysis. The actual number of prevalences is much greater. Splitting the main cohort into smaller, more homogeneous, subcohorts, could yield prevalences that are more personalized.

### Limitations

This study has some known limitations. The coverage analysis refers to only some of the symptoms and disorders, as explained in the Methods section. On one hand, because Kahun is an EBCIT and presently does not cover physical examination nor does it save personal information for follow-up visits, some valuable information was not covered in this tool by definition. Therefore, the full coverage of the NAMCS is unknown. On the other hand, many other symptoms and disorders that were not included in NAMCS exist in Kahun’s knowledge engine. Therefore, we recommend further coverage analysis of other tools and reference databases. Moreover, the NAMCS and DH cohort both cover the US population. Although the US population is diverse, some of the insights from this study are less relevant to other populations.

Although Kahun’s diagnostic ability showed encouraging results and improved performance compared with previous studies [13], no such tool can be considered perfect. Therefore, constant improvement of Kahun is advised, and as a result, the grounded prevalences suggested in this study will probably be refined in the future. Since the focus of this study was not on Kahun’s accuracy rate, the sample that was used to determine this accuracy rate included only about 10% of the medical vignette data (250/2550). Thus, further research that focuses on EBCITs’ diagnosis ability and includes more data should be considered in the future.

An additional limitation of the Kahun-DH cohort is that it consists only of patients who can afford DH services and actively seek medical care. This fact can potentially lead to selection bias and limit the generalizability of our real-world prevalences.

Moreover, since the setting of this cohort was based on digital encounters and included younger patients with fewer comorbidities, there might be an underrepresentation of older patients with more comorbidities, and the relevant prevalences might be affected. Therefore, future research involving typical primary care settings should be conducted.

Another limitation of this study is that most of the authors are employed by Kahun. This potential bias may have influenced the design and interpretation of the study results. However, we have taken steps to minimize the impact of this bias by using rigorous methods and statistical analyses and by involving other authors who are not employed by Kahun in analyzing some of the data. Despite these efforts, we recognize that the potential for bias remains and we encourage readers to carefully evaluate the evidence presented in this study and consider alternative perspectives.

### Comparison With Prior Work

As far as we know, only one study, conducted by Perlman et al [17], showed the potential advantage of using clinical intake tools to infer medical insights. Specifically, the study identified relative rates and associations between disorders and symptoms. That in-depth study included 71,619 self-assessments of COVID-19 participants. The study focused only on one disorder: COVID-19. Although it also included only several predetermined symptoms, it managed to identify prominent results such as an increased probability of having COVID-19 if a loss of smell or taste was experienced. It is worth noting that the demographic characteristics of the participants in that study, which were mainly females in their 30s, were similar to another study regarding clinical intake tools [14] and similar to our results.

In our view, the pioneering work done by Perlman et al [17] is extremely significant. Because their study had a relatively narrow scope in terms of coverage, symptoms, and disorders, we believed that at least one study with a wider scope was needed to support our main hypothesis. To the best of our knowledge, this study showed, for the first time, the potential of an EBCIT to generate prevalences of many symptoms in diseases.

### Conclusions

Kahun has the ability to cover most of the symptoms that patients may present while addressing most of the possible disorders. Although not perfect, Kahun has the potential to serve as a reliable medical database and provide plausible EBM diagnoses. Thus, increased use of EBCITs, such as Kahun, could help an improvement in EBCITs’ ability to uncover medical insights, including discovering and grounding prevalences of symptoms in disorders, which might support physicians in medical diagnosis and expand medical knowledge.

## Abbreviations

DH: digital health care
EBCIT: evidence-based clinical intake tool
EBM: evidence-based medicine
HIPPA: Health Insurance Portability and Accountability Act
ICD-10: International Statistical Classification of Diseases, Tenth Revision
NAMCS: National Ambulatory Medical Care Survey

## Data Availability

The data sets generated or analyzed during this study are available from the corresponding author upon reasonable request.
